# Identification of genetic variants and individual genes associated with postpartum hypocalcemia in Holstein cows

**DOI:** 10.1038/s41598-023-49496-1

**Published:** 2023-12-11

**Authors:** Larissa C. Novo, Michael B. Poindexter, Fernanda M. Rezende, José E. P. Santos, Corwin D. Nelson, Laura L. Hernandez, Brian W. Kirkpatrick, Francisco Peñagaricano

**Affiliations:** 1 Department of Animal and Dairy Sciences, University of Wisconsin, Madison, WI 53706 USA; 2 Department of Animal Sciences, University of Florida, Gainesville, FL 32611 USA

**Keywords:** Animal breeding, Genetic association study

## Abstract

Periparturient hypocalcemia is a complex metabolic disorder that occurs at the onset of lactation because of a sudden irreversible loss of Ca incorporated into colostrum and milk. Some cows are unable to quickly adapt to this demand and succumb to clinical hypocalcemia, commonly known as milk fever, whereas a larger proportion of cows develop subclinical hypocalcemia. The main goal of this study was to identify causative mutations and candidate genes affecting postpartum blood calcium concentration in Holstein cows. Data consisted of blood calcium concentration measured in 2513 Holstein cows on the first three days after parturition. All cows had genotypic information for 79 k SNP markers. Two consecutive rounds of imputation were performed: first, the 2513 Holstein cows were imputed from 79 k to 312 k SNP markers. This imputation was performed using a reference set of 17,131 proven Holstein bulls with 312 k SNP markers. Then, the 2513 Holstein cows were imputed from 312 k markers to whole-genome sequence data. This second round of imputation used 179 Holstein animals from the 1000 Bulls Genome Project as a reference set. Three alternative phenotypes were evaluated: (1) total calcium concentration in the first 24 h postpartum, (2) total calcium concentration in the first 72 h postpartum calculated as the area under the curve; and (3) the recovery of total calcium concentration calculated as the difference in total calcium concentration between 72 and 24 h. The identification of genetic variants associated with these traits was performed using a two-step mixed model-based approach implemented in the R package MixABEL. The most significant variants were located within or near genes involved in calcium homeostasis and vitamin D transport (*GC*), calcium and potassium channels (*JPH3* and *KCNK13*), energy and lipid metabolism (*CA5A*, *PRORP*, and *SREBP1*), and immune response (*IL12RB2* and *CXCL8*), among other functions. This work provides the foundation for the development of novel breeding and management tools for reducing the incidence of periparturient hypocalcemia in dairy cattle.

## Introduction

Periparturient hypocalcemia is a complex metabolic disorder that occurs at the onset of lactation because of a sudden irreversible loss of calcium incorporated into colostrum and milk. Some cows are unable to quickly adapt to this demand and succumb to clinical hypocalcemia, commonly known as milk fever, which affects around 5% of dairy cows^1^. A larger proportion of cows develop subclinical hypocalcemia which increases the risk of the cow to develop other peripartum diseases, such as retained placenta, uterine prolapse, metritis, displaced abomasum, ketosis, and mastitis^2^. The economic losses due to periparturient hypocalcemia are substantial due to on-farm death, premature culling, extended days open, and increased veterinary and treatment costs, among others^3^.

Periparturient hypocalcemia is a heritable trait with heritability estimates between 0.01 and 0.35 depending on the breed and the methodology^4^. The magnitude of these heritability estimates suggests that genetic selection can be effective to reduce the incidence of periparturient hypocalcemia in dairy cattle. Little is known, however, about the genes underlying cow’s susceptibility to periparturient hypocalcemia. Previous genomic studies in Holstein cows identified genes implicated in vitamin D metabolism, calcium, and potassium homeostasis, such as *GC*, *LRRC38*, *KCNK9*, and *CYP27A*^5,6^. In addition, Sasaki et al.^7^ reported genes *PKIB*, *DDIT4*, *PER1*, and *NUAK1*, as potential biomarkers for milk fever predisposition based on gene expression of peripheral blood mononuclear cells.

The identification of causal mutations and individual genes affecting periparturient hypocalcemia could have multiple benefits, including better understanding of the molecular mechanisms underlying this complex metabolic disorder, promote the development of new drugs, therapies, and prevention strategies, and contribute to the design of novel breeding strategies. As such, the aim of this study was to use imputed whole-genome sequence data to identify genetic variants associated with three alternative periparturient hypocalcemia traits, namely (1) total blood calcium concentration in the first 24 h postpartum, (2) total blood calcium concentration in the first 72 h postpartum, and (3) the difference in total blood calcium concentration between 72 and 24 h postpartum. We also characterized genes located near the most significant variants.

## Materials and methods

### Phenotypic and genotypic data

The data consisted of 2513 Holstein cows (938 primiparous and 1575 multiparous) with blood calcium concentrations measured on the first, second, and third day after parturition. The samples were collected in five different experiments performed in 2 different dairy herds in the US from December 2015 to June 2020. Records with blood calcium concentration ≥ 3.0 mmol/L were removed from the analysis because they were considered lab errors. After data editing, the mean (± SD) for blood calcium concentration was 2.13 (± 0.28) mmol/L, with a minimum and maximum of 0.68 and 2.99, respectively. All cows with blood calcium records had genotypic information for 79,060 SNP markers.

### Alternative postpartum hypocalcemia traits

**Figure 1:**
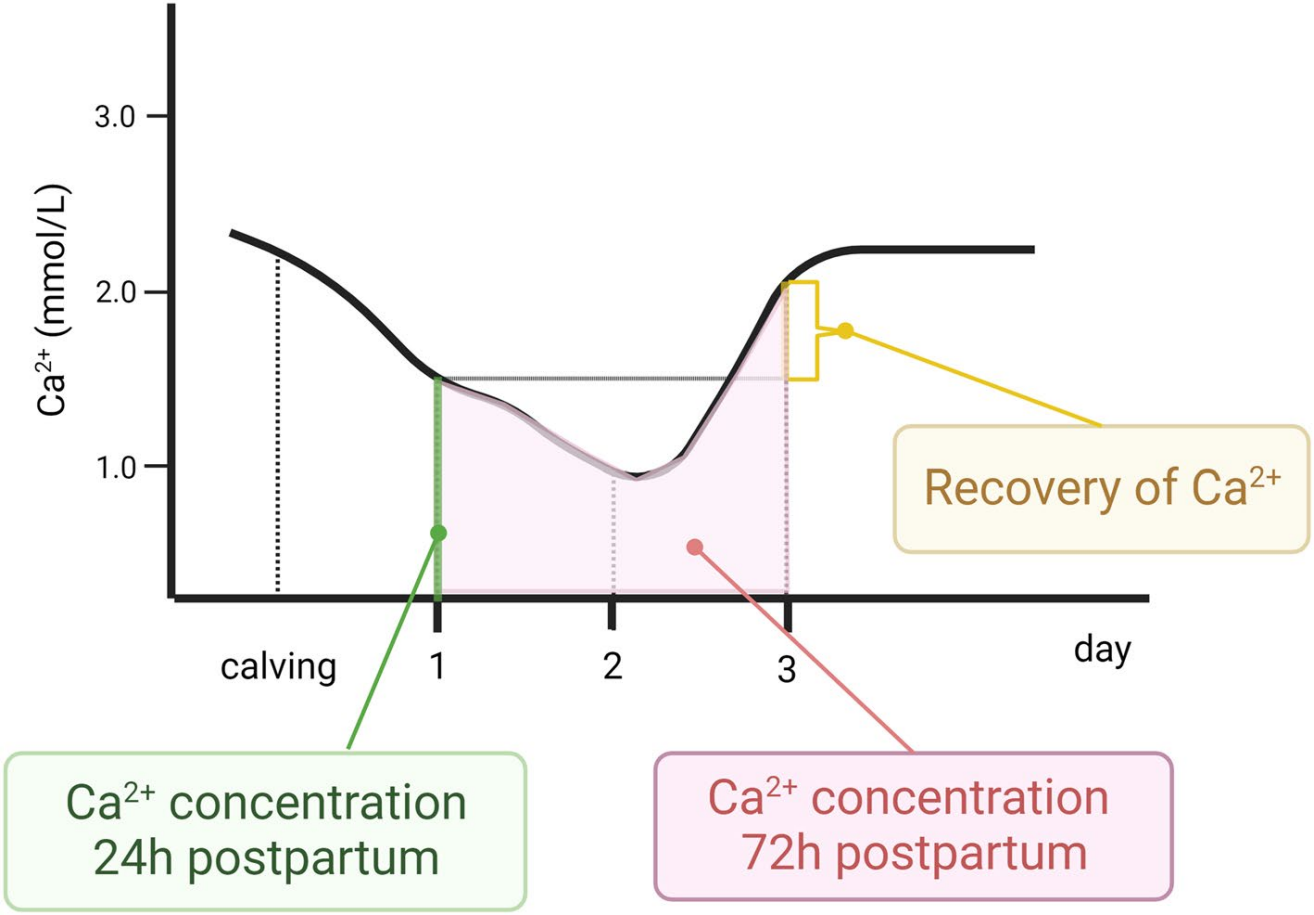
Three alternative phenotypes: total calcium concentration in the first 24 h postpartum, total calcium concentration in the first 72 h postpartum calculated as the area under the curve, and the recovery of calcium concentration calculated as the difference in calcium concentration between 72 and 24 h.

Three alternative postpartum blood calcium concentration traits were analyzed (Fig. 1): (1) total calcium concentration in the first 24 h postpartum (mmol/L), (2) total calcium concentration in the first 72 h postpartum calculated as the area under the curve (mmol/L) using the trapezoid function; and (3) the recovery of calcium concentration calculated as the difference in calcium concentration between 72 and 24 h (mmol/L). Table 1 shows the descriptive statistics for these three alternative postpartum hypocalcemia phenotypes.

**Table 1:** Descriptive statistics for three alternative postpartum blood calcium profiles.

Trait	N	Mean	Range
Total calcium concentration in the first 24 h postpartum, mmol/L	2513	2.03	0.82:2.98
Total calcium concentration in the first 72 h postpartum, AUC^1^	2513	4.56	1.76:8.96
Recovery of calcium concentration^2^	2513	0.21	−1.16:7.36

^1^AUC = area under the curve in mmol/L from 24 to 72 h postpartum.

^2^Difference in blood calcium concentration (mmol/L) between 72 and 24 h postpartum.

### Imputation process

The 2513 Holstein cows with postpartum blood calcium records and 79,060 SNP genotypes were imputed to whole-genome sequence in order to identify causal mutations associated with periparturient hypocalcemia. The imputation was performed in two steps, first from 79 k to 312 k markers, and then from 312 k markers to whole-genome sequence^8^. The 79 k and 312 k sets of SNP markers are both available in the BovineHD Genotyping BeadChip (Illumina Inc., San Diego, CA)^9^.

#### From 79 k to 312 k markers

The first imputation step aimed to provide a bridge between a medium density SNP chip and whole-genome sequence. As such, the 79 k SNP genotypes were imputed to 312,615 SNP genotypes using 17,131 Holstein bulls born between 1995 and 2008 as reference population. The imputation process was performed using FImpute3^10^ using the default parameters.

#### From 312 k markers to whole-genome sequence

After the first imputation step, the imputed 312 k markers were used as a new target for the imputation to whole-genome sequence. A total of 179 US Holstein bulls born in United States and part of the 1000 Bulls Genome project were used as reference panel^11^. The imputation was performed again using Fimpute3, and each chromosome was processed separately.

### Genomic scan

Only autosomal markers with a call rate > 0.9 and a minor allele frequency ≥ 1% for SNP densities 79 k and 312 k, and ≥ 0.01% for whole-genome sequence were retained for this analysis. After quality control, a total of 76,389, 300,621 and 11.6 million markers were available for the alternative genomic scans. The three alternative periparturient hypocalcemia phenotypes were analyzed using a two-step mixed-model-based approach^12^. Note that the following two steps were performed for each of the 3 phenotypes and each of the 3 SNP densities (9 runs in total).

In the first step, the following model was fitted:

1 $$\mathbf{y}=\mathbf{X}\mathbf{b}+\mathbf{Z}\mathbf{u}+\mathbf{e}$$

Where $\mathbf{y}$ is the vector of periparturient hypocalcemia records, $\mathbf{b}$ is the vector of fixed effects, $\mathbf{u}$ is the vector of random animal effects, and $\mathbf{e}$ is the vector of random residual effects. The fixed effects included herd-experiment-treatment (18 levels), lactation number with 5 levels (1 to 5 +) and calf category with 3 levels (male, females, twins). The incidence matrices $\mathbf{X}$ and $\mathbf{Z}$ relate phenotypic records to fixed and animal effects, respectively. The two random effects were assumed to follow a multivariate normal distribution with $\mathbf{u}\sim N\left(0,\mathbf{G}{\sigma }_{u}^{2}\right)$ and $\mathbf{e}\sim N\left(0,\mathbf{I}{\sigma }_{e}^{2}\right)$, where ${\sigma }_{u}^{2}$ and ${\sigma }_{e}^{2}$ are the animal additive genetic and residual variances respectively, $\mathbf{G}$ is the genomic relationship matrix, and $\mathbf{I}$ an identity matrix. For the genomic scan using whole-genome sequence, matrix $\mathbf{G}$ was created using 50,196 SNPs randomly selected across the entire genome^13^. The variance–covariance matrix for this first model was estimated as ${\mathbf{V}}_{0}= \mathbf{Z}\mathbf{G}{\mathbf{Z}}^{\prime}{\sigma }_{u}^{2}+\mathbf{I}{\sigma }_{e}^{2}$.

In the second step, the following model was fitted for every SNP:

2 $$\mathbf{y} = \mathbf{X}{\varvec{\upbeta}} + {X}_{SNP}{{\beta }_{SNP} +{\varvec{\epsilon}}}$$

where ${X}_{SNP}$ is the design matrix for the marker under consideration and ${\beta }_{SNP}$ is the regression coefficient, also known as SNP effect. This model assumes that ${\varvec{\epsilon}}\sim N\left(0,{\mathbf{V}}_{0}{\sigma }_{e}^{2}\right)$, where ${\mathbf{V}}_{0}$ is estimated in the first step. The significance of each SNP effect was evaluated using the following test statistic:

$$\mathbf{z}= \frac{{{\mathbf{X}}^{\prime}}_{SNP}{\mathbf{V}}_{0}^{-1}(\mathbf{y}-\mathbf{X}\widehat{\upbeta })}{\sqrt{{{\mathbf{X}}^{\prime}}_{SNP}{{\varvec{V}}}_{0}^{-1}{\mathbf{X}}_{SNP}}}$$

which approximates the Wald test, and hence, is asymptotically standard normal. These analyses were performed using the R package MixABEL^12^ (version 0.1–3). The *P*-values were adjusted for multiple comparisons using the Benjamini–Hochberg procedure^14^. Statistical significance was declared using an adjusted *P*-values smaller than 0.05.

The assignment of significant genetic variants to bovine genes was based on the latest bovine genome reference ARS-UCD 1.2 using Ensembl^15^. Genetic variants located upstream, downstream, or within annotated genes were considered.

## Results

### Total calcium concentration in the first 24 h postpartum

**Figure 2:**
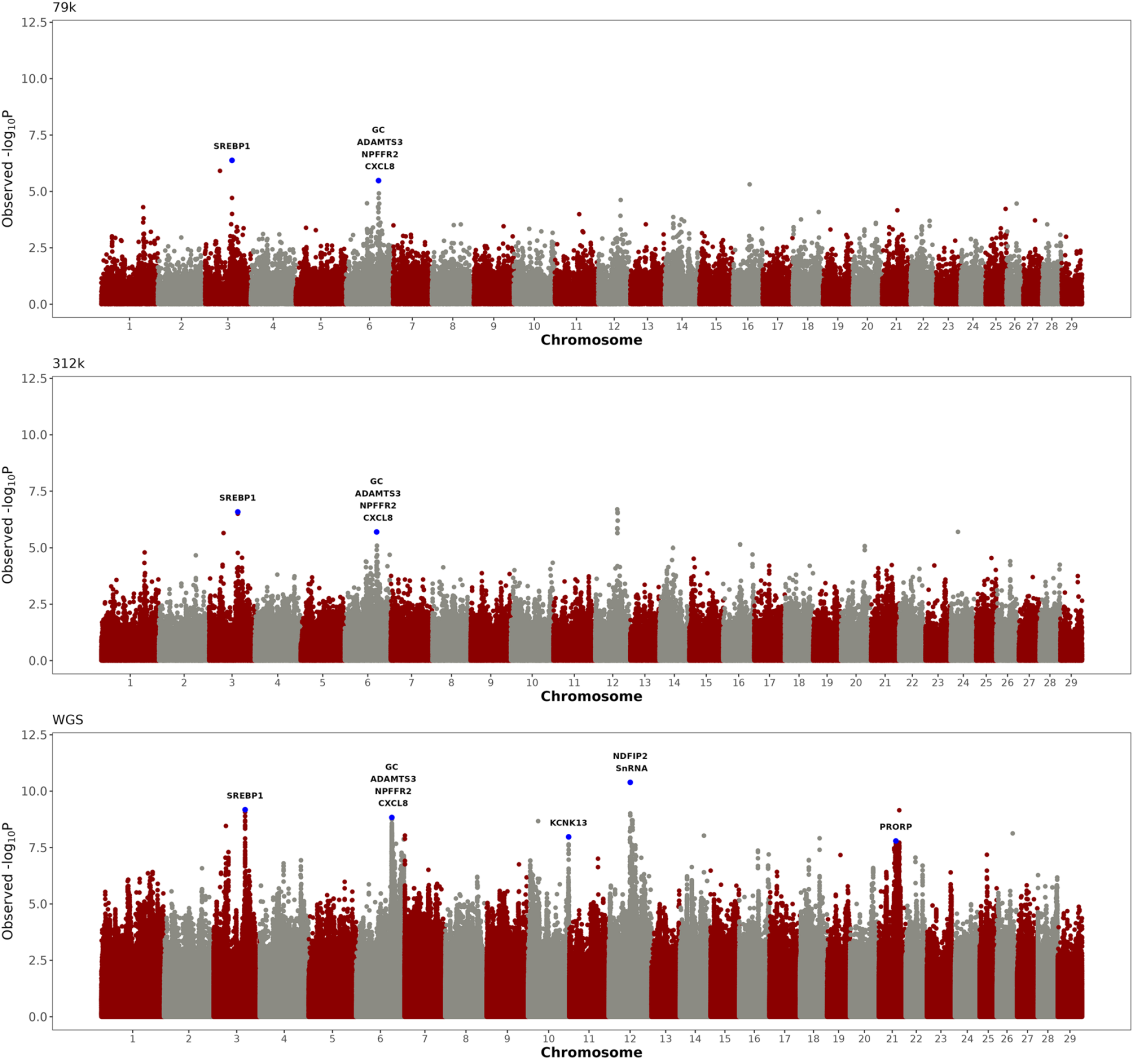
Genomic scans for total blood calcium concentration measure in the first 24 h postpartum. The scans were performed using three different SNP densities, namely 79 k SNP, 312 k SNP, and whole-genome sequence (WGS). Genes directly implicated in calcium homeostasis, calcium and potassium channels, energy metabolism, and immune response, are highlighted in the Manhattan plots.

The heritability estimate for total calcium concentration in the first 24 h postpartum was 0.186. Five different genomic regions located on BTA3, BTA6, BTA10, BTA12 and BTA21 showed significant associations with calcium concentration in the first 24 h postpartum (Fig. 2, Table 2). The most significant variant on BTA3 is upstream to the gene *SREBP1*, a major gene implicated in lipid metabolism. The most significant variant on BTA6 is localized between the genes *GC* and *NPFFR2*, which are implicated in vitamin D signaling pathway and regulation of MAPK cascade, respectively. This genomic region on BTA6 also harbors gene *CXCL8*, which encodes for a pro-inflammatory cytokine, and gene *ADAMTS3*, which encodes a protease involved in the biosynthesis of collagen. Interestingly, both peaks on BTA3 and BTA6 were also detected using 79 k and 312 k SNP chips. Moreover, the most significant variant on BTA10 is located in an intron of gene *KCNK13*, a two-pore domain potassium channel that is regulated by extracellular calcium concentrations. The most significant variant on BTA12 is located upstream gene *NDFIP2*, which is involved in metal ion transport and positive regulation of protein ubiquitination. Finally, the most significant variant on BTA21 is located in an intron of gene *PRORP*, a member of the mitochondrial ribonuclease P complex involved in mitochondrial tRNA 5′-end processing. The major peaks detected on BTA10, BTA12, and BTA21 were detected only using whole-genome sequence data.

**Table 2:** Most significant variants and candidate genes associated with three alternative postpartum blood calcium concentration traits.

Chr	Pos	*P*-value	Gene	Location	Process
Total calcium concentration in the first 24 h postpartum					
3	77.8	6.6e−10	*SREBP1*	Upstream	lipogenesis
6	87.2	1.5e−09	*GC*	Upstream	vitamin D transport
6	87.2	1.5e−09	*NPFFR2*	Inside	regulation MAPK cascade
6	87.2	1.5e−09	*ADAMTS3*	Downstream	biosynthesis of collagen
6	88.8	6.6e−07	*CXCL8*	Upstream	pro inflammatory cytokine
10	101.7	8.2e−07	*KCNK13*	Inside	potassium channel
12	54.5	4.1e−11	*NDFIP2*	Upstream	protein ubiquitination
21	45.5	1.6e−08	*PRORP*	Inside	energy metabolism
Area under the curve of total calcium concentration in the first 72 h postpartum					
3	77.9	8.8e−08	*SREBP1*	Upstream	lipogenesis
3	78.0	4.8e−08	*IL12RB2*	Inside	interleukin 12 receptor
6	87.2	1.0e−06	*GC*	Upstream	vitamin D transport
11	94.8	1.0e−06	*DENND1A*	Inside	membrane trafficking
12	18.9	1.6e−07	*CAB39L*	Downstream	calcium-binding protein
12	18.8	1.3e−06	*FNDC3A*	Inside	RNA binding activity
18	13.2	4.9e−08	*JPH3*	Inside	calcium ion transport
18	13.4	1.4e−06	*CA5A*	Inside	one-carbon metabolism
18	13.4	1.4e−06	*BANP*	Upstream	regulation of cell cycle
18	22.7	2.6−e06	*IRX3*	Upstream	energy homeostasis
26	34.8	5.9e−07	*CCDC186*	Inside	response to bacterium
Recovery of calcium concentration^1^					
9	63.6	4.6e−18	*SYNCRIP*	Upstream	mRNA metabolism
9	63.6	4.6e−18	*SNX14*	Upstream	intracellular trafficking

^1^Difference in blood calcium concentration between 72 and 24 h postpartum.

### Area under the curve of total calcium concentration in the first 72 h postpartum

**Figure 3:**
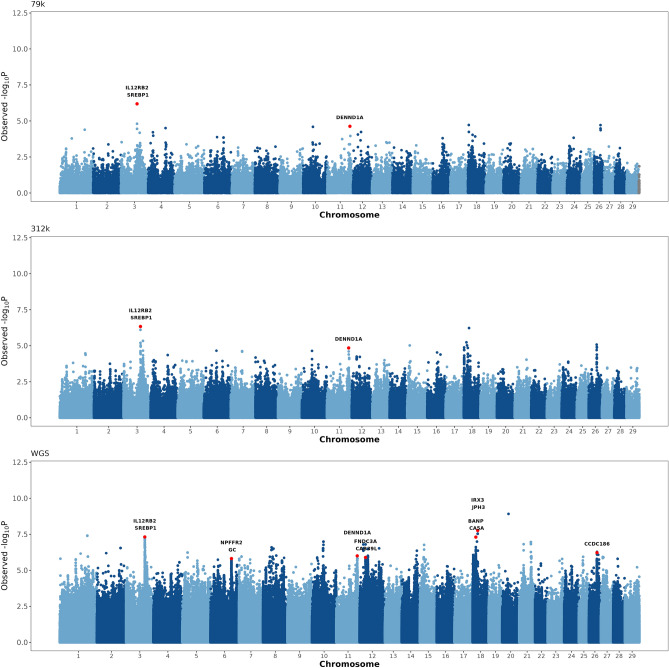
Genomic scans for total blood calcium concentration measure in the first 72 h postpartum. The scans were performed using three different SNP densities, namely 79 k SNP, 312 k SNP, and whole-genome sequence (WGS). Genes directly implicated in calcium homeostasis, calcium and potassium channels, energy metabolism, and immune response, are highlighted in the Manhattan plots.

The heritability estimate for the area under the curve of total calcium concentration in the first 72 h postpartum was 0.135. Six genomic regions located on BTA3, BTA6, BTA11, BTA12, BTA18, and BTA26 showed significant associations with the area under the curve of total calcium concentration in the first 72 h postpartum (Fig. 3, Table 2). The major peaks on BTA3 and BTA6, which harbor genes *SREBP1* and *GC*, respectively, were also identified as significantly associated with calcium concentration in the first 24 h postpartum. The most significant variant on BTA11 is located in an intron of gene *DENND1A*, a member of the connecdenn family, which is involved in endocytosis and protein transport. Two significant regions were detected on BTA12, one region harbors gene *CAB39L*, which encodes for a calcium-binding protein involved in energy stress, and the other region harbors gene *FNDC3A*, a transmembrane protein with RNA binding activity. The most significant region on BTA18 harbors several genes, including *JPH3*, *IRX3*, *BANP* and *CA5A*. The genes are implicated in calcium ion transport into cytosol, energy homeostasis, regulation of cell cycle, and one-carbon metabolism.

### Recovery of calcium concentration

**Figure 4:**
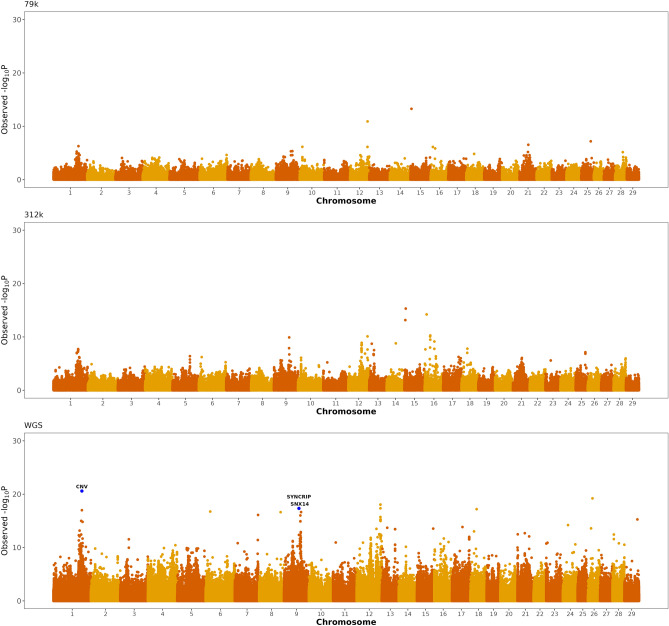
Genomic scans for the recovery of blood calcium concentration calculated as the difference in calcium concentration between 72 and 24 h. The scans were performed using three different SNP densities, namely 79 k SNP, 312 k SNP, and whole-genome sequence (WGS). Genes implicated in mRNA metabolism, transport, and intracellular trafficking are highlighted in the Manhattan plots.

The heritability estimate for recovery of calcium concentration was 0.043. Three genomic regions located on BTA1, BTA9 and BTA12 showed significant associations with recovery of blood calcium concentration from first to third day postpartum (Fig. 4, Table 2). The most significant variant on BTA1 is located in a copy number variant segment previously associated with the immune process. The most significant variant on BTA9 is upstream genes *SYNCRIP* and *SNX14*. Gene *SYNCRIP* encodes a member of the heterogeneous nuclear ribonucleoprotein family, RNA binding proteins that regulate alternative splicing, polyadenylation, and other aspects of mRNA metabolism and transport. Gene *SNX14* encodes a member of the sorting nexin family which is implicated in intracellular trafficking. The significant region on BTA12 has no genes currently annotated.

## Discussion

We investigated the genetic basis of three interrelated postpartum blood calcium traits, namely calcium concentration in the first 24 h postpartum, area under the curve for total calcium concentration in the first 72 h postpartum, and the recovery of calcium concentration calculated as the difference in calcium concentration between 72 and 24 h. Periparturient hypocalcemia is a complex metabolic disorder, and the use of alternative phenotypes enables to capture different nuances in changes in postpartum blood calcium. We used the area under the curve for total calcium concentration in the first 72 h postpartum as a way to quantify total calcium in the first 3 days postpartum. We acknowledge that different patterns of blood calcium in the first 72 h postpartum can yield the same area under the curve. In fact, there is no single trait that perfectly describes the pattern of calcium concentration, and that is why we decided to evaluate simultaneously three interrelated postpartum blood calcium traits. Neves and collaborators have shown that the association of blood calcium concentration with cow performance varies depending on the timing of assessment during the early postpartum period^16,17^.

Some of the most significant variants are located near or within genes directly implicated in calcium homeostasis. Indeed, one of the regions highly associated with calcium concentration in the first 24 h and also the first 72 h harbors gene *GC*. This gene encodes the vitamin D binding protein, which is responsible for the transport of most vitamin D3 metabolites in the plasma^18^. Among vitamin D3 metabolites, 25-hydroxyvitamin D3 is the main circulating form in the plasma, and is converted into 1,25-dihydroxyvitamin D3, the biologically active vitamin D metabolite, in the kidney. When there is a decrease of blood calcium concentration, the parathyroid gland responds with increased secretion of parathyroid hormone, which upregulates renal production of 1,25-dihydroxyvitamin D3. The 1,25-dihydroxyvitamin D3 increases blood calcium and phosphorus, in part by stimulating bone resorption, renal reabsorption and gastrointestinal absorption of calcium^19,20^. The 1,25-dihydroxyvitamin D3 also has potent immunomodulatory activity, and it is worth noting that variants associated with the gene *GC* were implicated in mastitis of dairy cows^21^.

The genomic scans identified genes that are involved in calcium and potassium channels. For instance, gene *JPH3*, associated with total calcium concentration in the first 72 h postpartum, encodes a member of the junctophilin family which is implicated in the physical approximation of plasmalemmal and sarcoplasmic/endoplasmic reticulum membranes. Junctophilins, such as *JPH3*, facilitate signal transduction in excitable cells between plasmalemmal voltage-gated calcium channels and intracellular calcium release channels^22^. Gene *KCNK13*, associated with calcium concentration in the first 24 h postpartum, encodes a potassium channel containing two pore-forming domains. This channel is regulated by different factors, including arachidonic acid, halothane, and high extracellular calcium concentrations^23^.

Genes implicated in energy metabolism were found to be associated with postpartum blood calcium concentration. For instance, gene *CA5A*, associated with area under the curve for total calcium concentration in the first 72 h postpartum, encodes a member of carbonic anhydrases, a large family of zinc metalloenzymes that catalyze the reversible hydration of carbon dioxide and participate in a variety of biological processes, including respiration, calcification, acid–base balance, and bone resorption. *CA5A* is localized in the mitochondria, it is expressed primarily in the liver, and it is involved in one-carbon metabolic process^24^. Gene *PRORP*, also known as *MRPP3*, associated with calcium concentration in the first 24 h postpartum encodes a mitochondrial RNA processing enzyme within the rNase P complex that is implicated in mitochondrial energy metabolism. Recent studies have shown that mutations on *PRORP* can reduce mitochondrial calcium, which in turn reduces insulin release from the pancreatic islet β cells. Note that reduced insulin secretion results in decreased insulin concentrations which contributes to imbalanced metabolism and insulin resistance^25^. The region on BTA3 that showed significant associations with calcium concentration in the first 24 h and also 72 h postpartum harbors gene *SREBP1*. This gene encodes a transcription factor that binds to the sterol regulatory element 1, a DNA motif that is found in the promoter of the low-density lipoprotein receptor gene and other genes involved in sterol biosynthesis. Note that there is a close link between negative energy balance and hypocalcemia in periparturient dairy cows. Indeed, negative energy balance has been demonstrated to decrease circulating calcium while the lack of intracellular calcium impairs carbohydrate metabolism, exacerbating the negative energy balance state^26^.

Periparturient cows experience significant immune dysregulation, and because intracellular calcium signaling is important for immune cell activation, the development of periparturient hypocalcemia contributes to periparturient immune suppression^27^. Interestingly, we found several genes associated with postpartum blood calcium that are directly implicated in the immune response. For instance, gene *IL12RB2*, associated with area under the curved for total calcium concentration in the first 72 h postpartum, encodes a subunit of the interleukin 12 receptor complex and plays an important role in Th1 cell differentiation^28^. Gene *CXCL8*, associated with calcium concentration in the first 24 h postpartum, encodes interleukin 8, a member of the CXC chemokine family and a major mediator of the inflammatory response. Interleukin 8 is secreted by mononuclear macrophages, neutrophils, eosinophils, and T lymphocytes, among others, and it functions as a chemotactic factor by guiding immune cells to the site of infection^29^. Gene *CCDC186*, associated with area under the curved for total calcium concentration in the first 72 h postpartum, is not yet well characterized but it appears it enables small GTPase binding, and it is involved in insulin secretion and response to bacterium.

Of special interest, the most significant variants associated with postpartum blood calcium traits are all located in non-coding regions, either upstream or downstream genes or within intronic regions. Causative mutations in non-coding regions such as regulatory elements (enhancers, insulators, and promoters) and untranslated regions (5’UTR and 3’UTR) typically affect the level of gene expression, whereas mutations on introns could affect both level of gene expression but also the structure of the protein. Interestingly, The Encyclopedia of DNA Elements (ENCODE) project, launched as a follow-up to the Human Genome Project with the goal of identifying and annotating functional elements of the human genome, revealed that many DNA variants associated with diseases lie within non-coding elements^30^.

## Conclusions

We performed a comprehensive genomic analysis of three alternative postpartum blood calcium concentration traits in dairy cattle. The most significant variants were located within or near genes involved in calcium homeostasis and vitamin D transport, calcium and potassium channels, energy and lipid metabolism, and immune response, among other functions. These findings can contribute to the development of novel breeding and management strategies for reducing periparturient hypocalcemia in dairy cattle.

## Data Availability

The phenotypes and genotypes analyzed in this study were obtained from River Ranch Dairy (Hanford, CA) and the Council on Dairy Cattle Breeding (Bowie, Maryland), respectively. These datasets were used under agreement, and hence, are not publicly available. However, data are available upon request to River Ranch Dairy (Jack de Jong, jdjwrivran@aol.com) and the Council on Dairy Cattle Breeding (Joao Dürr, joao.durr@uscdcb.com).
